# Inhalation of hydrogen gas attenuates airway inflammation and oxidative stress in allergic asthmatic mice

**DOI:** 10.1186/s40733-018-0040-y

**Published:** 2018-03-15

**Authors:** Ning Zhang, Changwen Deng, Xingxing Zhang, Jingxi Zhang, Chong Bai

**Affiliations:** 1 Department of Naval Aeromedicine, The Second Military Medical University, Shanghai, 200433 China; 2 Department of Respiratory and Critical care medicine, Changhai Hospital, the Second Military Medical University, Shanghai, 200433 China

**Keywords:** Asthma, Oxidative stress, Hydrogen gas inhalation, Cytokine, Pulmonary function

## Abstract

**Background:**

Asthma is a worldwide common chronic airway disease that cannot be cured and results in the huge burden in public health. Oxidative stress was considered an important mechanism in the pathogenesis of asthma. Hydrogen gas been demonstrated to function as a novel antioxidant and exert therapeutic antioxidant activity in a number of diseases and the function of this nontoxic gas in asthma was unclear. The purpose of the study aims to examine the effect of inhalation hydrogen gas on the pathophysiology of a mouse model of asthma.

**Methods:**

A murine model of ovalbumin (OVA)-induced allergic airway inflammation was used in this study. Briefly, Mice were sensitized to ovalbumin and received inhalation of 67% high concentration of hydrogen gas for 60 min once a day for 7 consecutive days after OVA or PBS challenge respectively. Lung function was assessed in the apparatus with 4 channels of biological signal system. Morphology and goblet cell hyperplasia were stained by H/E and Periodic acid-Schiff staining. Cytologic classification in the bronchial alveolar lavage fluid (BALF) was analyzed by Wright Giemsa staining. Serum, BALF and lung tissue were collected for biochemical assay. One-way analysis of variance (ANOVA) was used to determine statistical significance between groups. Multiple comparisons were made by Bonferroni’s Multiple Comparison Test by using GraphPad Prism 5 software.

**Results:**

Inhalation of hydrogen gas abrogated ovalbumin-induced the increase in lung resistance. Concomitantly, the asthmatic mice showed severe inflammatory infiltration and goblet cell hyperplasia which were reversed by hydrogen gas inhalation. Hydrogen gas inhalation reduced significantly the number of total cells, eosinophils and lymphocytes in BALF. Increased level of IL-4, IL-13, TNF-α and CXCL15 in the BALF and IL-4 in the serum were decreased significantly after inhalation. Hydrogen gas inhalation markedly upregulated the activity of decreased superoxide dismutase and significantly attenuated the increased level of malondialdehyde and myeloperoxidase.

**Conclusions:**

Hydrogen gas inhalation improves lung function and protects established airway inflammation in the allergic asthmatic mice model which may be associated with the inhibition of oxidative stress process. This study provides a potential alternative therapeutic opportunity for the clinical management of asthma.

## Background

Asthma is a common chronic respiratory disease with increased prevalence, resulting in a heavy burden on public health world wide. This challenging disease characterized by persistent airway inflammation cannot be cured. Although many efforts have been made to increase the therapeutic effect. Oxidative stress plays an important role in the pathogenesis of this chronic disorder. Inflammation induces lung oxidative stress reaction and leads to a large number of reactive oxygen species [1]. The effect of reactive oxygen species on the pathogenesis of asthma is to stimulate pulmonary function impairment, mast cell degranulation, airway remodelling and mucus secretion by epithelium, all of which in turn can aggravate the local inflammation of the lung.

Hydrogen is considered an inert gas and has been used in medical applications to prevent decompression sickness in deep divers [2]. It was reported in 2007 that hydrogen delivered via inhalation has authentic antioxidant and anti-apoptotic properties that can protect the brain against ischaemia/reperfusion injury by selectively neutralizing hydroxyl radicals [3]. This report aroused considerable interest worldwide. The therapeutic effects of molecular hydrogen on various diseases have been investigated regarding its antioxidation capability [4] and its anti-inflammation [5] and anti-apoptosis [6] capabilities. Compared with traditional antioxidants, hydrogen is a small molecule that can easily dissipate throughout the body and cells, and it is sufficiently mild that it does not disturb metabolic oxidation-reduction reactions or ROS-mediated cell signalling. Thus, it may be a safe and effective antioxidant for pulmonary diseases. As the mainstream administration route, inhalation is considered the preferred methods in the treatment of asthma. Recently, accumulating evidence has demonstrated various types of diseases involving oxidative stress, including ischaemic heart disease [7], stroke [8], acute lung injury [9] and inflammatory bowel disease [5], benefitted from or were protected by inhalation of hydrogen gas. The effect of this kind of gas on asthma is not fully understood. Therefore, the aim of this study was to investigate the anti-inflammation and antioxidation function of inhalation of high concentrations of hydrogen gas in a mouse asthmatic model.

## Methods

### Animals

BALB/c mice purchased from the Experimental Animal Centre of Second Military Medical University were housed in rooms maintained at constant temperature (21 ± 2 °C) and humidity (55 ± 15%) with a 12-h light/dark cycle and allowed food and water ad libitum. Female mice at 6–8 weeks of age were used for experiments. Forty mice were randomly divided into four groups with 10 mice each: sham control group (Control, C), asthma group (A), hydrogen-gas treatment group (AH), and hydrogen gas control group (Hydrogen, H). The experiment was repeated three times. Experimental protocols were approved by the Ethical Committee for Animal Studies of Second Military Medical University, Shanghai, China.

### Ovalbumin (OVA) sensitization/aerosol challenge

**Fig. 1:**
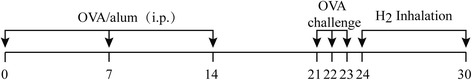
The protocol for the hydrogen gas inhalation experiments in the asthmatic mouse model

Mouse models of asthma were prepared with chicken OVA sensitization and challenge. Briefly, 20 μg (100 μl) chicken OVA that was emulsified with alum (2.25 mg Al(OH)_3_, 2 mg Mg(OH)_2_; Pierce, USA) was intraperitoneally injected into mice on Days 0, 7 and 14. The airway challenge was provided by an aerosol of saline alone or 1% (0.01 g/ml) OVA in saline generated by ultrasonic nebulization (DeVilbiss, USA), which the mice inhaled for 20 min on Days 21, 22, and 23. The mice in the AH and H groups were treated by inhaling a high concentration of hydrogen gas (67%) for 60 min once a day for 7 consecutive days after OVA and PBS challenge, respectively (Fig. 1). The mice were anaesthetized with an overdose of chloral hydrate i.p. followed by exsanguination 24 h after the last treatment.

### Hydrogen gas administration

The mixed gas consisting of 67% H_2_ and 33% O_2_ was produced by the AMS-H-01 hydrogen oxygen nebulizer (Asclepius, Shanghai, China), which was specifically designed to extract the hydrogen and oxygen from water. The mice were placed into a transparent closed box (20× 18 × 15 cm, length x width x height) into which the mixed gas was introduced at a rate of 200 ml/min throughout the experiments. The box was flushed with mixed gases for 30 min to replace the air in the box. During each experiment, the concentration of hydrogen gas in the box was monitored by Thermal trace GC ultra-gas chromatography (Thermo Fisher, MA, USA).

### Measurement of pulmonary function

Pulmonary function was assessed in the apparatus with 4 channels of biological signal system (Model SMUP-PC, Fudan University). Mice were anaesthetized i.p. with hydrazine hydrate, tracheotomised with a blunted 18-gauge cannula and ventilated. Gas flow was determined and recorded by the Fleisch air flow transducer connected with trachea intubation under condition of spontaneous mouse breathing. The pressure inside the oesophagus can be recorded continuously as chest pressure. Lung resistance (RL) and dynamic compliance of the respiratory system (Cdyn) were calculated by the Amdur and Mead method according to respiratory rate, tidal volume, respiration flow and chest pressure [10].

### Tissue collection and preparation

Bronchoalveolar lavage (BAL) was performed after airway hyperresponsiveness measurements when the animal was still under anaesthesia. A total volume of 0.5 ml cold PBS was used to lavage the lungs three times. Blood was collected by cardiac puncture with a 20-gauge needle, and serum was prepared through centrifugation in serum tubes and stored at − 20 °C for further analyses. The mice were euthanized with cervical dislocation. The left lung lobe was removed and fixed in 4% formaldehyde overnight. The superior and middle lobes of the right lung were snap frozen on dry ice and stored at − 80 °C until further processing. Protein was extracted from the snap-frozen samples of pulmonary tissue with a cell lysis kit (Bio-plexTM Cell Lysis kit, Bio-Rad) supplemented with proteinase inhibitors (Sigma-Aldrich).

### Assessment of lung histology

Following appropriate preparation, 5-μm thick paraffin sections of the left lung were stained with haematoxylin and eosin (H&E) to evaluate general morphology. The degrees of lung inflammation from five airway sections randomly distributed throughout the left lung were evaluated by one analyst blinded to the groups using a subjective scale ranging from 0 to 4 (0, normal; 1, mild; 2, moderate; 3, severe; 4, more severe). Periodic acid-Schiff (PAS) staining was applied to detect muco-substances. Images of lung tissues with airways were captured by a Nikon microscope. PAS-positive area and total area of corresponding bronchial epithelium were calculated, and the adopted grading system was: 0, no goblet cells; 1, < 15%; 2, 15–30%; 3, 30–45%; 4, 45–60%; 5, > 60% [11].

### Oxidative stress index in lung tissue

A portion of the tissue preparation was made from right lung tissue that was homogenized with normal saline, and the preparation was centrifuged at 2500 rpm at 4 °C for 10 min. The resultant supernatant was equilibrated with saline and used to determine the levels of proteins in the tissue homogenates. The levels of superoxide dismutase (SOD), glutathione (GSH), catalase (CAT), malondialdehyde (MDA), myeloperoxidase (MPO), and 8-hydroxydeoxyguanosine (8-OHdG) were determined using commercial kits (Nanjing Jiancheng, Nanjing, China). The determinations were performed strictly according to the manufacturer’s instructions.

### Cytokine in bronchial alveolar lavage fluid (BALF) and serum

Enzyme-linked immunosorbent assay (ELISA) (R&D Systems, Minneapolis, MN, USA) was used to determine the expression levels of IL-4, IL-5, IL-13, IL-6, TNF-α and CXCL15 in BALF and serum according to the manufacturers’ instructions. The absorbance was measured at 450 nm using a microplate reader (Model 680; Bio-Rad, Hercules, CA, USA).

### Statistical analysis

The GraphPad Prism 5 software was used for statistical analysis. Data from all the mice in each group were presented as the means ± SD or the median (inter quartile range [IQR]) for continuous variables. One-way analysis of variance (ANOVA) was used to determine statistical significance between groups. Multiple comparisons were made by Bonferroni’s Multiple Comparison Test. *P* < 0.05 was considered statistically significant.

## Results

### Hydrogen gas inhalation decreased lung resistance in the asthmatic mice model

**Fig. 2:**
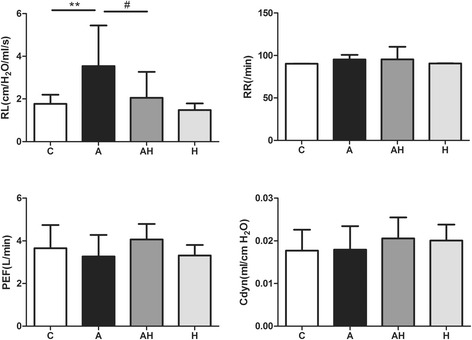
The effect of hydrogen gas inhalation on lung function in the asthmatic mouse model. C: control group (*n* = 10), A: asthma group (*n* = 10), AH: asthma plus hydrogen gas inhalation group (*n* = 10), H: control plus hydrogen gas inhalation group (*n* = 10). **: vs control group, *P* < 0.01; #: vs asthma group, *P* < 0.05

Lung resistance (RL) increased in the asthmatic mouse model (3.537 ± 1.9 cm/H_2_O/ml/s vs 1.765 ± 0.43 cm/H_2_O/ml/s, *P* < 0.01) compared with the control group and was significantly lower in the AH group compared with the A group (2.052 ± 1.2 cm/H_2_O/ml/s vs 3.53 ± 1.9 cm/H_2_O/ml/s, *P* < 0.05) (Fig. 2). There was no significant difference in the respiratory rate (RR), peak flow rate (PEF) and dynamic compliance (Cdyn) (Fig. 2) among the four groups.

### Hydrogen gas inhalation improved the histology and mucus production in the asthmatic mouse model

**Fig. 3:**
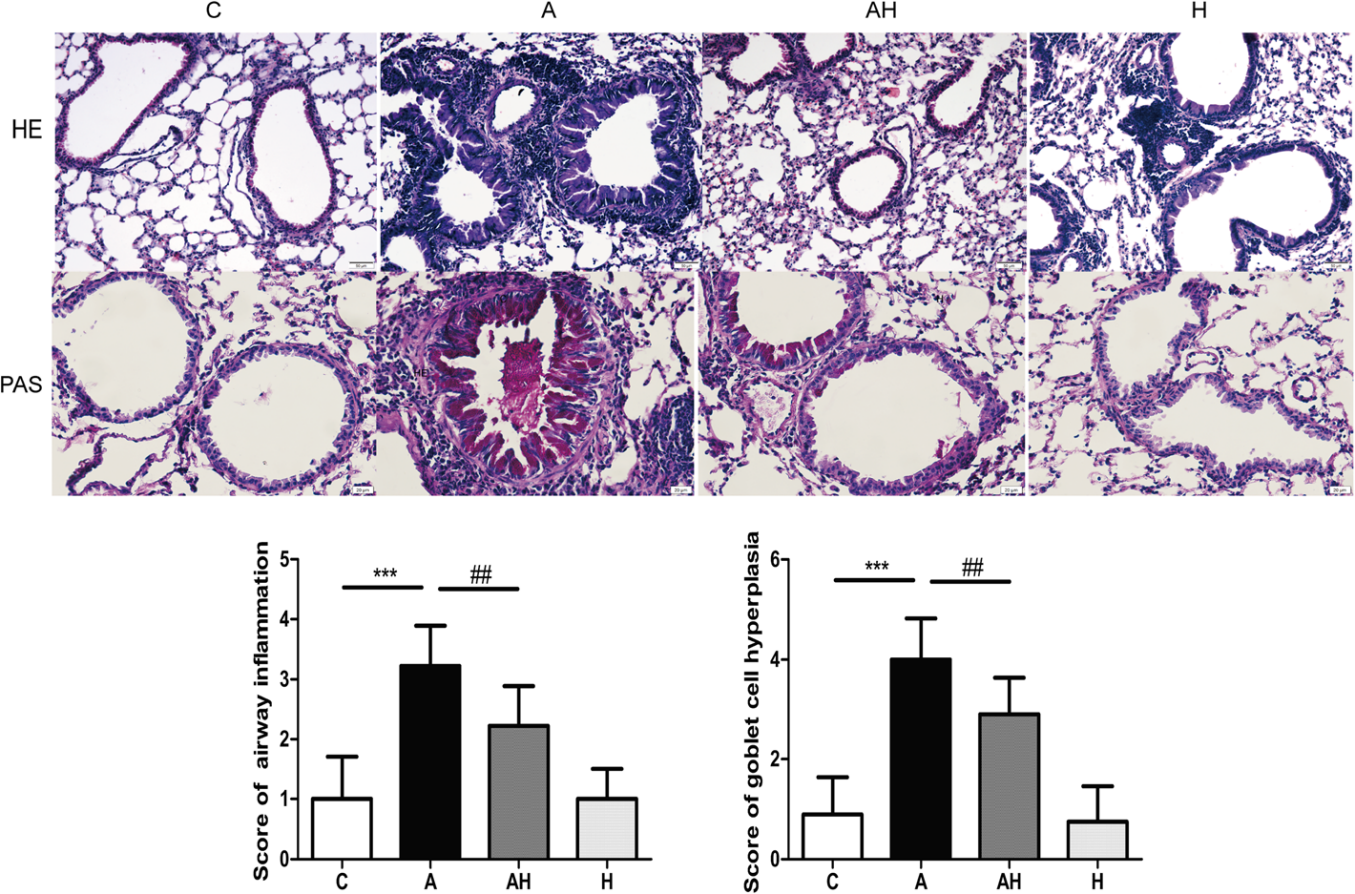
Morphologic findings and scores of bronchial wall in control animals (C, *n* = 10), asthmatic mice model (A, *n* = 10) and asthmatic mice model with hydrogen gas inhalation (AH, *n* = 10). Staining with haematoxylin-eosin (H&E) solution, periodic acid-Schiff (PAS); magnification 40×)

Mouse models exhibited cardinal histopathological signs of human asthmatic lungs, including peribronchial and perivascular inflammatory infiltrates, with most notably eosinophilia (3.22 ± 0.67 versus 1.00 ± 0.74, *P* < 0.001), goblet cell hyperplasia (4.00 ± 0.81 versus 0.90 ± 0.73, *P* < 0.001), airway wall thickening and airway obstruction (Fig. 3) compared with the control group. Hydrogen gas inhalation attenuated this accumulation of inflammatory cells (2.22 ± 0.67, *P* < 0.01) and reduced the epithelial goblet cell hyperplasia (2.9 ± 0.73, *P* < 0.01) (Fig. 3).

### Hydrogen gas inhalation reduced the levels of inflammatory cells in bronchoalveolar lavage fluid from asthmatic mice model

**Fig. 4:**
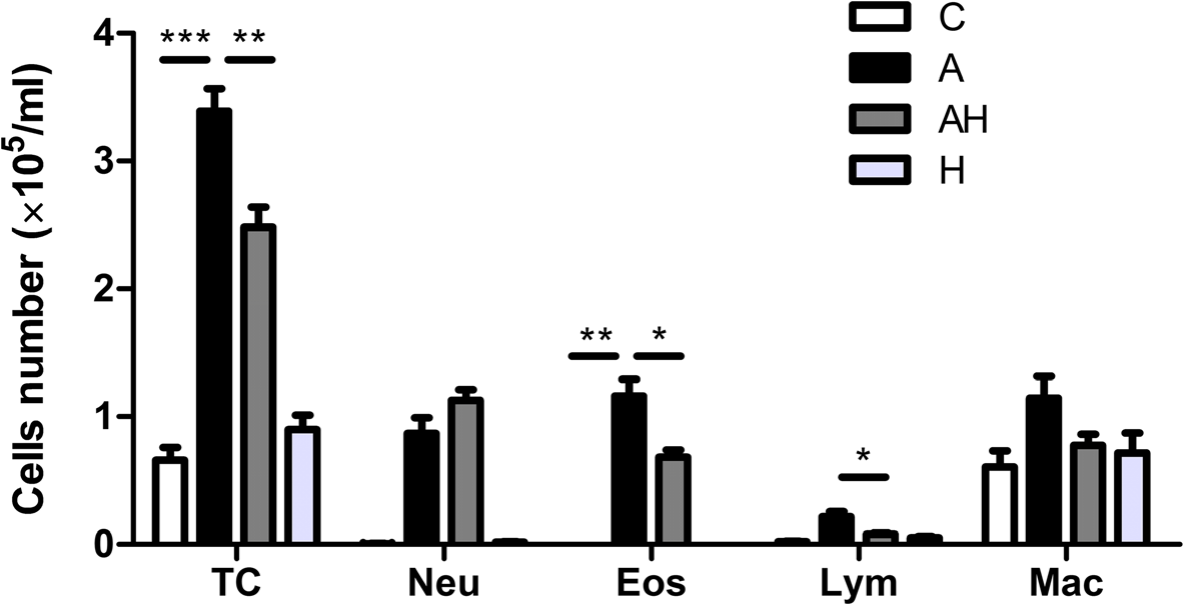
The number of total cells, neutrophils, eosinophils, lymphocytes and macrophages in the BALF in control animals (C, *n* = 10), asthmatic mouse model (A, *n* = 10), asthmatic mice with hydrogen gas inhalation (AH, *n* = 10) and control animals with hydrogen gas inhalation (H, *n* = 10). Statistical comparison between groups was performed using analysis of variance followed by Tukey’s test. * *P* < 0.05, ** *P* < 0.01, *** *P* < 0.001 compared to the control group, # *P* < 0.05, ## *P* < 0.01 compared to the asthma group

There was a significant increase in the number of total cells ((3.39 ± 0.56) × 10^5^/ml), *P* < 0.001), neutrophils (0.86 ± 0.39) × 10^5^/ml, *P* < 0.001), eosinophils (1.16 ± 0.4) × 10^5^/ml, *P* < 0.001), lymphocytes (0.21 ± 0.13) × 10^5^/ml,* P* < 0.001) and macrophages (1.14 ± 0.53) × 10^5^/ml, *P* < 0.001) in BALF of asthmatic mice models compared with those of controls ((0.66 ± 0.3) × 10^5^/ml, (0.006 ± 0.003) × 10^5^/ml, (0.001 ± 0.003) × 10^5^/ml, (0.02 ± 0.009) × 10^5^/ml, (0.6 ± 0.31) × 10^5^/ml, respectively). Hydrogen gas inhalation resulted in significant reduction in the number of total cells ((2.48 ± 0.51) × 10^5^/ml), *P* < 0.001), eosinophils (0.68 ± 0.18) × 10^5^/ml, *P* < 0.001), and lymphocytes (0.07 ± 0.03) × 10^5^/ml, *P* < 0.001), and a nonsignificant decrease in the number of macrophages compared with asthmatic mouse models. The pure hydrogen gas inhalation had no effect on the BALF cell numbers (Fig. 4).

### Hydrogen gas inhalation attenuated the elevated levels of inflammatory cytokines present in BALF from the asthmatic mouse model

**Fig. 5:**
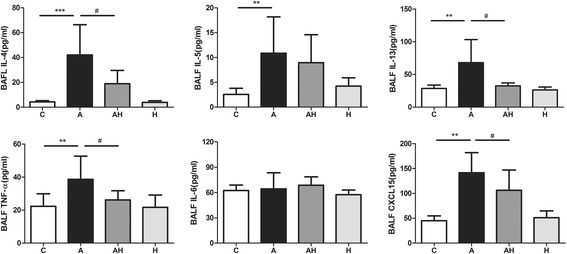
The concentration of inflammatory cytokines in the BALF in control animals (C, *n* = 10), asthmatic mouse model (A, *n* = 10), asthmatic mice with hydrogen gas inhalation (AH, *n* = 10) and control animals with hydrogen gas inhalation (H, *n* = 10). * *P* < 0.05, ** *P* < 0.01, *** *P* < 0.001 compared to the control group, # *P* < 0.05 compared to the asthma group

There was a significant increase in IL-4 (42.11 ± 24.31 pg/ml, *P* < 0.001), IL-5 (10.85 ± 7.33 pg/ml, 0.01), IL-13 (68.04 ± 35.26 pg/ml, *P* < 0.01), TNF-α (38.62 ± 14.12 pg/ml, *P* < 0.01) and CXCL15 (141.4 ± 40.75 pg/ml, *P* < 0.01) in BALF of asthmatic mouse models compared with those of controls (4.24 ± 1.08 pg/ml, 2.55 ± 1.25 pg/ml, 28.48 ± 5.37 pg/ml, 22.28 ± 7.57 pg/ml, and 44.92 ± 9.95 pg/ml, respectively). Hydrogen gas inhalation resulted in significant reductions in the concentrations of IL-4 (18.91 ± 10.66 pg/ml, *P* < 0.05), IL-13 (32.57 ± 4.43 pg/ml, *P* < 0.05), TNF-α (26.12 ± 5.59 pg/ml, *P* < 0.05) and CXCL15 (106.3 ± 40.75 pg/ml, *P* < 0.05). Hydrogen gas inhalation nonsignificantly decreased the expression of IL-5 (8.97 ± 5.62 pg/ml) in BALF. There was no effect on the IL-6 expression in BALF in the asthmatic mouse model with or without hydrogen gas inhalation. The pure hydrogen gas inhalation had no effect on the levels of inflammatory cytokines in BALF (Fig. 5).

### Hydrogen gas inhalation attenuated the elevated levels of inflammatory cytokines present in serum from an asthmatic mouse model

**Fig. 6:**
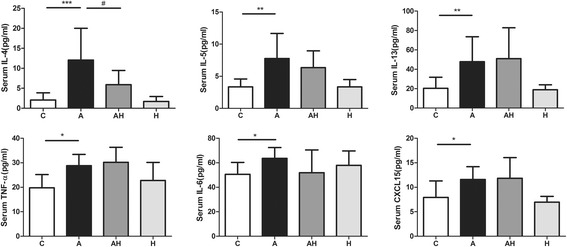
The serum concentration of inflammatory cytokines in control animals (C, *n* = 10), asthmatic mouse model (A, *n* = 10), asthmatic mice with hydrogen gas inhalation (AH, *n* = 10) and control animals with hydrogen gas inhalation (H, *n* = 10). * *P* < 0.05, ** *P* < 0.01, *** *P* < 0.001 compared to the control group, # *P* < 0.05 compared to the asthma group

There was a significant increase in IL-4 (12.06 ± 7.93 pg/ml, *P* < 0.001), IL-5 (7.64 ± 3.91 pg/ml, 0.01), IL-13 (47.77 ± 25.81 pg/ml, *P* < 0.01), TNF-α (28.80 ± 4.59 pg/ml, *P* < 0.05), IL-6 (63.56 ± 8.88 pg/ml, *P* < 0.05) and CXCL15 (11.58 ± 2.62 pg/ml, *P* < 0.05) in BALF of asthmatic mouse models compared with those of controls (2.07 ± 1.81 pg/ml, 3.35 ± 1.21 pg/ml, 20.37 ± 11.43 pg/ml, 19.79 ± 5.39 pg/ml, 50.56 ± 9.69 pg/ml and 7.92 ± 3.38 pg/ml, respectively). Hydrogen gas inhalation resulted in a significant reduction in the concentration of IL-4 (5.92 ± 3.53 pg/ml, *P* < 0.05). Hydrogen gas inhalation had no significant effect on the serum concentration of IL-5 (6.34 ± 2.62 pg/ml), IL-13 (50.97 ± 31.84 pg/ml), TNF-α (30.17 ± 6.16 pg/ml) and CXCL15 (11.82 ± 4.24 pg/ml) in the asthmatic mouse model with or without hydrogen gas inhalation. The pure hydrogen gas inhalation had no effect on the levels of inflammatory cytokines in the serum (Fig. 6).

### Hydrogen gas inhalation attenuated the oxidative stress index presented in lung homogenates from asthmatic mouse models

**Fig. 7:**
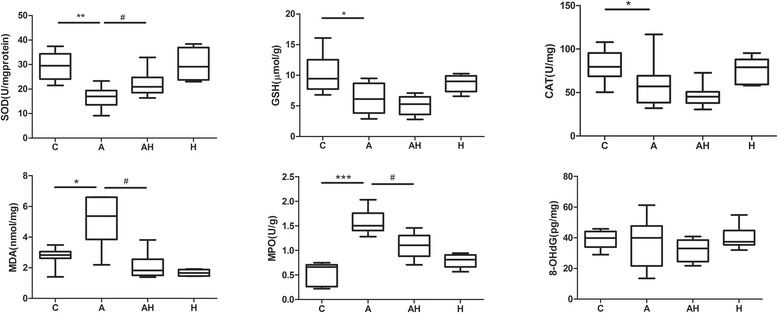
The levels or activities of SOD, MDA, GSH, CAT, MPO, and 8-OHdG of lung tissue in control animals (C, *n* = 10), asthmatic mouse model (A, *n* = 10), asthmatic mice with hydrogen gas inhalation (AH, *n* = 10) and control animals with hydrogen gas inhalation (H, *n* = 10). * *P* < 0.05, ** *P* < 0.01, *** *P* < 0.001 compared to the control group, # *P* < 0.05 compared to the asthma group

The levels or activities of SOD, MDA, GSH, CAT, MPO, and 8-OHdG in lung tissue were determined to assess the hydrogen gas inhalation in protection from oxidative damage (Fig. 7). The levels of MDA (5.37 (3.85–6.59) nmol/mg) and MPO (1.51 (1.41–1.76) U/g) increased significantly (*P* < 0.05), and the levels of SOD activity (16.98 (13.5–19.39) U/mg)), GSH (6.13 (3.83–8.70) μmol/g), and CAT (57.02 (38.3–69.30) U/mg) decreased significantly (*P* < 0.05) in the lung tissues of the asthmatic mouse group compared with those of controls (2.82 (2.62–3.05) nmol/mg, 0.66 (0.27–0.71) U/g, 29.53 (24.08–34.43) U/mg, 9.45 (7.76–12.54) μmol/g and 79.61 (68.74–95.39) U/mg, respectively). A significant reduction of MDA (1.82 (1.50–2.56) nmol/mg, *P* < 0.05) and MPO levels (1.11 (0.89–1.31) U/g, *P* < 0.05) was observed, and a significant increase in SOD activities (20.92 (18.53–24.76) U/mg) was observed in the asthmatic mice with hydrogen gas inhalation group (*P* < 0.05). However, the levels of GSH, CAT and 8-OHdG did not change significantly after hydrogen gas inhalation.

## Discussion

From the present study, we conclude that inhalation of hydrogen gas protects against asthma in mouse models by improving lung function, ameliorating mucus production and decreasing inflammation and oxidative stress markers.

Asthma is a chronic inflammatory airway disease whose pathogenesis is not completely elucidated. However, the “airway injury from free radicals and oxidant/antioxidant imbalance” theory has aroused widespread attention. Oxidative stress plays an important role in the occurrence and development of bronchial asthma, especially in the acute exacerbation period [12, 13]. Excessive production of oxidative stress has been reported to lead to airway inflammation, lung function decline, mucus overproduction, tissue injury, and remodelling in animal models and human studies [14, 15]. Fatani found that MDA increased in asthmatic patients, especially in the exacerbation periods [16]. The mouse model used in this study presented lung resistance increase, different types of inflammatory cell infiltration dominated by eosinophils, and mucus plug formation. All these are similar to the manifestations observed in the acute asthma attack [17]. We also found oxidative marker elevation and antioxidative enzyme reduction, confirming that oxidative stress exists in this classic asthmatic mouse model.

Hydrogen is a colourless and odourless gas composed of the simplest molecule in the world. Molecular hydrogen functions as an antioxidant and anti-inflammatory agent [18]. The routes of hydrogen gas administration in animal models and human clinical studies are roughly classified into three types: inhalation of hydrogen gas, drinking hydrogen dissolved in water and injection of hydrogen dissolved in saline [19]. Recently, molecular hydrogen gas was proved to be an effective pathway to carry out the antioxidative treatment. A number of studies demonstrated the protective effects of hydrogen on various inflammation-involved diseases, such as oxygen toxicity induced lung injury [20], lung ischemia-reperfusion injury [21], and COPD [22]. Our study first indicated that molecular hydrogen could improve the allergic asthmatic morphologic changes by inhalation.

Chronic airway inflammation mediated by Th2-oriented cytokines was the most important characteristic in allergic asthma. The oxidative stress injury involves the initiation and progression of inflammatory cascades, which consequently elevate the levels of inflammatory cytokines. Inhibition of inflammation might provide a benefit for the protection of asthma. In our study, we found the hydrogen gas inhalation significantly alleviated the pathologic inflammation degree and mucus content in the lung tissue. Meanwhile, hydrogen gas inhalation decreased the level of the typical Th2-type cytokines IL-4 and IL-13 in BALF and/or in serum; these cytokines are the two major mediators responsible for the eosinophil recruitment, airway function decrease and mucus hypersecretion. Inhibition of these cytokines could be used to partially explain the simultaneous airway resistance decrease and pathophysiologic inflammatory cell accumulation in pulmonary tissues and BALF. Chemokines have also been linked to asthmatic pathogenesis. TNF-α elevation was associated with increased airway resistance, recruitment of neutrophils, eosinophils and monocytes, and even airway remodelling. Mouse CXCL15, a chemokine represented by IL-8 in humans, was found to recruit neutrophils to inflammatory areas. Inhalation of hydrogen gas could also have an inhibitory effect on TNF-α and CXCL15, suggesting that this method will have potential effects on neutrophil infiltration.

Imbalance of excessive production of oxidative stress and antioxidative capacity resulted in oxidative-related injury. MDA is an important indicator of lipid peroxidation and oxidative conditions. In the allergic asthmatic mouse model, increased MDA showed a partial decrease after hydrogen gas inhalation. Yu et al. found that hydrogen-rich saline improved the serum MDA and SOD levels in an allergic rhinitis animal model [23] involved with suppression of eotaxin, further confirming that molecular hydrogen had protective effects on allergic disorders. MPO, which is a haemoprotein secreted during activation of neutrophils [24], was considered to stimulate the production of MDA and participate in neutrophilic airway inflammation in asthma [25]. The simultaneous decrease of MDA and MPO may suggest the interaction between these two substances.

Studies had revealed the suppressed activity of key antioxidant enzymes, including catalase, superoxide dismutase and glutathione peroxidase, in patients with bronchial asthma [26, 27]. Ahmad et al. also showed that the antioxidant enzymes (SOD and CAT) are found at lower levels in asthmatic patients [28]. In animal models, the decreased activity of SOD and CAT in the lungs of animals injected and inhaled with OVA supported the hypothesis that asthma was related to the reduction of antioxidative capacity [29]. This study revealed that hydrogen gas could reverse the decrease of SOD in the lungs of asthmatic mice. However, hydrogen gas had no effect on the other antioxidative enzymes, such as CAT, GSH and 8-hydroxy-2′-deoxyguanosine (8-OHdG), suggesting that hydrogen gas inhalation protected oxidative stress with limitations.

From this study, we also noted that pure hydrogen gas inhalation had no significant effect on lung function, inflammatory mediators and oxidative production, suggesting that inhalation is a safe method for application. Inhalation is a convenient method for clinical use, with no abnormal odour and no significant side effects.

There are some limitations of our study. We have not located the exact lower limit of space volume for the safe use of the mixed gas inhalation. The molecular mechanism underlying the protection needs to be further investigated.

## Conclusions

In summary, we demonstrated that inhalation of hydrogen gas may play a role in attenuating airway inflammation and improving lung function in an asthmatic mouse model. This protection may be involved with correcting the oxidative/antioxidative imbalance and suppressing inflammatory mediators. The result confirmed the biological effects of hydrogen and put forward a new and promising method for the treatment of this common chronic airway disease. More clinical trials are needed to prove the clinical safety of its use and the protective effects of hydrogen gas at the bedside.
